# Bacterial analysis in the early developmental stages of the black tiger shrimp (*Penaeus monodon*)

**DOI:** 10.1038/s41598-020-61559-1

**Published:** 2020-03-17

**Authors:** Pacharaporn Angthong, Tanaporn Uengwetwanit, Sopacha Arayamethakorn, Panomkorn Chaitongsakul, Nitsara Karoonuthaisiri, Wanilada Rungrassamee

**Affiliations:** 1 Microarray Research Team, National Center for Genetic Engineering and Biotechnology, 113 Thailand Science Park, Phahonyothin Road, Khlong Luang, Pathum Thani 12120 Thailand; 2 Shrimp Genetic Improvement Center (SGIC), National Center for Genetic Engineering and Biotechnology, Surat Thani, 84110 Thailand

**Keywords:** Microbial ecology, Next-generation sequencing

## Abstract

Microbial colonization is an essential process in the early life of animal hosts—a crucial phase that could help influence and determine their health status at the later stages. The establishment of bacterial community in a host has been comprehensively studied in many animal models; however, knowledge on bacterial community associated with the early life stages of *Penaeus monodon* (the black tiger shrimp) is still limited. Here, we examined the bacterial community structures in four life stages (nauplius, zoea, mysis and postlarva) of two black tiger shrimp families using 16S rRNA amplicon sequencing by a next-generation sequencing. Although the bacterial profiles exhibited different patterns in each developmental stage, *Bacteroidetes*, *Proteobacteria, Actinobacteria* and *Planctomycetes* were identified as common bacterial phyla associated with shrimp. Interestingly, the bacterial diversity became relatively stable once shrimp developed to postlarvae (5-day-old and 15-day-old postlarval stages), suggesting an establishment of the bacterial community in matured shrimp. To our knowledge, this is the first report on bacteria establishment and assembly in early developmental stages of *P. monodon*. Our findings showed that the bacterial compositions could be shaped by different host developmental stages where the interplay of various host-associated factors, such as physiology, immune status and required diets, could have a strong influence.

## Introduction

The shrimp aquaculture industry is one of the key sectors to supply food source to the world’s growing population. According to Food and Agriculture Organization (FAO), the black tiger shrimp (*Penaeus monodon)* production is one of the most important traded shrimp species due to their higher market demand and market price. Nonetheless, the domestication of the black tiger shrimp is not sustainable as its production has been facing difficulties owing to disease outbreaks and poor growth performance. Gut microbiota are known to critically influence their host physiology, metabolism and immunity in humans and animal models, e.g. mouse, fruit fly, zebra fish^1–4^. Their profound effects on the host^5,6^ provide potential applications to improve shrimp production. However, the knowledge on host-microbiota is still limited in non-model animals, particularly *P. monodon*. To response to this lack of fundamental knowledge on bacterial community structures, our research team has recently initiated efforts to determine intestinal bacteria in *P. monodon* and the interactions with their host. We have deciphered bacterial diversities from shrimp intestinal samples from different farm locations at the juvenile stage^7^, growing stages (15-day post larvae, 1-, 2-, and 3-month-old juveniles)^8^ covering different habitats in adult shrimp (wild and domestication)^9^.

In addition to determining bacterial community structures, we have previously reported that the ability to restore normal gut microbiome correlates with pathogen resistance in shrimp^10^. The effects of pathogens on intestinal microbiota of the two economically important shrimp species, *P. monodon* and *Litopenaeus vannamei* (the Pacific white shrimp) were investigated under the same rearing environment and diet. The bacterial profiles indicated that the presence of pathogenic *Vibrio harveyi* could alter the intestinal bacterial patterns differently in the two shrimp species. *L. vannamei*, which resisted the pathogens better than *P. monodon*, were able to re-establish their bacterial population to resemble those observed in the unexposed shrimp control group. *P. monodon*, on the other hand, have lost their ability to restore their bacterial balance. Our recent findings provide one of the first insights that intestinal bacterial population, altered by the presence of pathogen in shrimp intestines and intestinal bacterial stability, might contribute to the colonization resistance against the invading pathogens. Hence, intestinal microbial ecology management can potentially benefit disease prevention in aquaculture.

Taking into account of the importance of gut microbiome from other animals and our previous studies in shrimp, we believe that the understanding of microbiome will pave the way for disease control and sustainable shrimp farming. Several pieces of evidence have pointed that the bacterial community and individual bacteria species can play a role in larval survival with respect to a quality assessment and shrimp larval development, particularly the development of the intestinal immunity^11^. Several probiotics applications have shown promising results in reducing mortality. Their mode of actions is likely related to immune modulation and/or antagonistic inhibition of pathogenic organisms^12^. However, screening of those potential probiotics is currently performed using traditional screening methods, in which bacterial isolates will be tested for desired phenotypes (such as pathogen inhibition or ability to digest nutrients) or supplemented into the test diet prior to disease challenges. Lacking the knowledge of bacterial community structure, the pre- and probiotics development for aquatic organisms has to rely solely on this “trial and error” basis and eventually indeed becomes “a never-ending story”^13^.

To be able to develop pre- and probiotics effectively, an understanding of the baseline community since the early life stages of shrimp is necessary. Particularly, newly hatched shrimp possess a sterile, immature digestive system before they further mature into nauplius, zoea, mysis, and postlarvae^14^. After the nauplius stage, *P. monodon* begin to feed on microalgae or live feeds, which permit bacteria from external sources to start to flourish in the host intestine^15^. In other animals, the interactions between the host immunity and non-pathogenic bacteria during the early life stages is crucial for the further development of immunity^4,16,17^. Such interaction is deemed to also exist in shrimp because their immune system during early life in hatchery is often still developing to their full potentials. Therefore, the early life stages will be a prime period when the application of feed additives, such as probiotics, could help establish healthy gut bacteria. To further advance this area, our study was aimed to characterize the baseline bacterial community associated with the early life stages of *P. monodon*. This understanding will serve as a key basis that galvanizes the sustainable development of effective shrimp feed or management practice to exert protective effects in hatchery.

## Results

### Distribution of taxa and phylotypes

**Figure 1:**
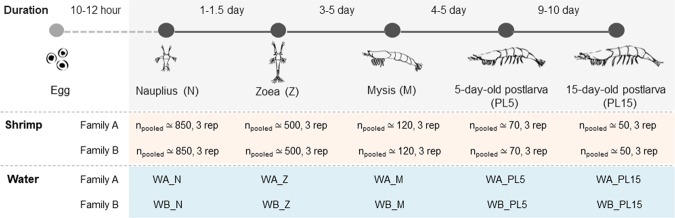
Schematic diagram of collection of early developmental stages in black tiger shrimp. Shrimp and rearing water (W) were collected at the stages of nauplius (N), zoea (Z), mysis (M), 5-day-old postlarva (PL5) and 15-day-old postlarva (PL15) from different family (Family A and Family B) for microbiota analysis.

To determine bacteria associated with *P. monodon* at early developmental stages, shrimp from four life stages (nauplius (N), zoea (Z), mysis (M), 5- and 15-day-old postlarva (PL5 and PL15, respectively) along with their corresponding rearing water were collected from two families (Family A and B) for 16S amplicon sequencing using Illumina platform (Fig. 1). After filtering for high quality sequences, 16S rRNA sequences were assigned into operational taxonomic units (OTUs) at 97% sequence similarity level, and further classified to a genus level based on the Ribosomal Database Project (RDP) (Table S1). The total number of OTUs ranged from 629 to 3,433, with an average (± standard deviation) of 2,350 ± 835 OTUs per sample. For rearing water, the total number of OTUs ranged from 2,005 to 3,177, with an average of 2,670 ± 424 OTUs. The average numbers of bacteria in taxonomical orders at different stages were in similar ranges (Table S1).

**Figure 2:**
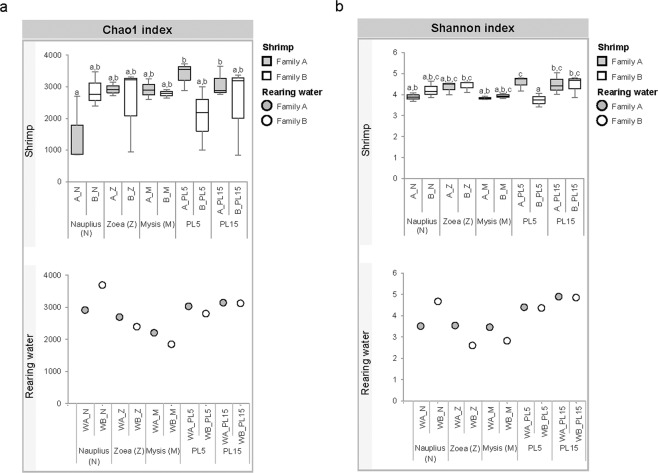
Diversity analysis. Chao1 index (**a**) and Shannon index (**b**) were used to estimate bacterial diversity of shrimp and rearing water at stages of nauplius (N), zoea (Z), mysis (M), 5-day-old postlarva (PL5) and 15-day-old postlarva (PL15) from different family (Family A and Family B) as shown with gray and white plot graph, respectively). The box delimits the 25^th^ and 75^th^ percentile, the line in each box indicates the median, and the whiskers indicate the lowest and highest values. Different letters show significant difference between groups by ANOVA (*p* value < 0.05) (n = 3).

To determine the coverage and bacterial richness, rarefaction curve and alpha diversity indices (Chao 1 and Shannon) were analyzed for the bacterial community associated with each life stage together with its rearing water. Our rarefaction analysis did not completely reach a plateau, indicating that the sequencing did not reach the saturation (Supplementary Fig. S1). Nevertheless, the Good’s coverage values were spanning from 0.82 to 1.00 with an average of 0.87 ± 0.06 that implied a sufficient coverage of bacterial richness for each community (Table S1). Consistently, Chao1 values were higher than the observed OTUs in all life stages and water, suggesting that further sequencing efforts will be necessary to increase the coverage (Fig. 2a). Shannon indices for four life stages with values ranging between 3.76 to 4.47 were not significantly different (*p* value < 0.05), indicating that all shrimp stages shared a similar spectrum of bacterial diversity (Fig. 2b).

### Establishment of bacterial community associated with shrimp at the early life stages

**Figure 3:**
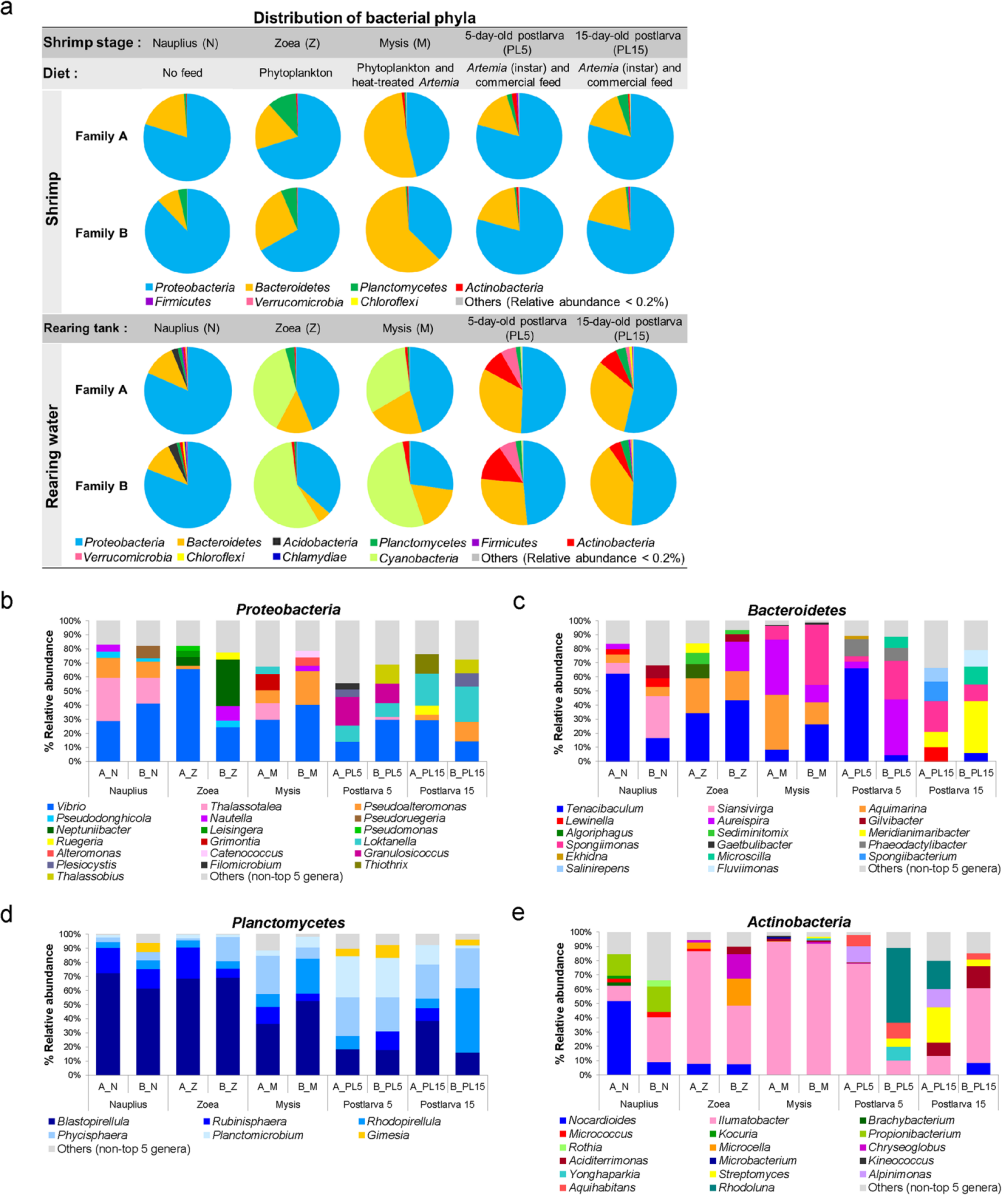
Dominant bacterial communities associated with shrimp and rearing water. Shrimp samples and rearing water were collected at nauplius (N), zoea (Z), mysis (M), 5-day-old postlarval (PL5) and 15-day-old postlarval (PL15) stages from two families (Family A and Family B). Distribution of bacterial phyla associated to shrimp and their rearing water with their relative abundance greater 0.2% were shown and those with their abundance less than 0.2% were grouped as other phyla. (**a**) Stacked bar plots represent the top 5 dominant bacterial genera associated with shrimp from each family at each life stage from each phylum, *Proteobacteria* (**b**), *Bacteroidetes* (**c**), *Planctomycetes* (**d**) and *Actinobacteria*. (**e**) Non-top five genera were shown under other genera.

We investigated the diversity of bacterial communities with respect to the early life stages of shrimp and their rearing water (Fig. 3). During early developmental stages of shrimp Family A and B, the major phyla associated with *P. monodon* were *Proteobacteria*, *Bacteroidetes*, *Planctomycetes* and *Actinobacteria* (Fig. 3a). Taxonomic analysis revealed that *Proteobacteria* was the most prevalent phylum found in all shrimp stages with the exception of the mysis stage in which *Bacteroidetes* was the dominating phylum. The relative abundance of *Planctomycetes* in zoea was higher than their presence in other stages, whereas *Actinobacteria* was most prevailing in the PL5 stage. The major bacterial phyla in all rearing water samples were dominated by *Proteobacteria*, *Bacteroidetes*, *Planctomycetes* and *Actinobacteria* during cultivation. Although *Proteobacteria* and *Bacteroidetes* were the most prevalent phyla found in all rearing water samples, the analysis of water collected during the zoea and mysis stages unveiled that the dominant phylum has shifted to *Cyanobacteria*, which were associated with phytoplankton given as a main feed diet in these stages (Fig. 3a). Interestingly, the relative abundance of *Actinobacteria* in rearing water of PL5 and PL15 was higher than other stages, and this was consistent with a bacterial pattern identified in shrimp. Our observations show that there were dynamic interactions observed among the host shrimp, microbiota and their rearing environments. To further validate the bacterial abundance, real-time PCR of shrimp and rearing water samples using specific primers to *Gammaproteobacteria*, *Alphaproteobacteria*, *Bacteroidetes*, *Actinobacteria* and *Firmicutes* showed a coherent relative abundance of bacterial patterns with the results obtained from the next-generation sequencing (Supplementary Fig. S2).

The five most abundant genera from those common phyla, *Proteobacteria*, *Bacteroidetes*, *Planctomycetes* and *Actinobacteria*, were compared in shrimp at each life stage (Fig. 3b–e). For instance, *Vibrio* was predominantly present in shrimp in all four stages (Fig. 3b). Bacterial genera previously reported to be associated with phytoplankton such as *Neptuniibacter* were found specifically in zoeae, which fed solely on phytoplankton. Similarly, *Artemia-*associated bacterial genera *Alteromonas* and *Nautella* were found predominantly in mysis which fed on *Artemia*. In addition, *Grimontia* was exclusively found at this stage. During the postlarval stage, *Thalassobius, Plesiocystis* and *Loktanella* were identified in both PL5 and PL15 shrimp while *Granulosicoccus* was present only in the former group.

Within phylum *Bacteroidetes*, genus *Tenacibaculum* was predominant in nearly all stages (Fig. 3c). Some genera exhibited stage-specific presence as shrimp matured from one stage to the other. For example, *Siansivirga* was found predominant in nauplii, whereas *Aquimarina* and *Aureispira* were mostly associated with zoeae and mysis shrimp. The presence of *Phaeodactylibacter* and *Ekhidna* was correlated only with PL5 shrimp while *Meridianimaribacter, Spongiibacterium, Salinirepens* and *Fluviimonas* were identified in PL15. The genus distribution patterns within *Planctomycetes* were similar across all stages in which *Blastopirellula*, *Rubinisphaera*, *Rhodopirellula*, *Phycisphaera* and *Planctomicrobium* were the predominant genera (Fig. 3d), yet *Gimesia* only later emerged to be associated with nauplii and postlarvae. Within phylum *Actinobacteria*, *Ilumatobacter* was found in all stages, whereas *Nocardioides* was classified in nauplii, zoeae and PL15 (Fig. 3e). *Kocuria, Propionibacterium, Brachybacterium* and *Rothia* were specific to the nauplius stage. Lastly, *Rhodoluna, Aquihabitans* and *Alpinimonas* were found uniquely in the postlarval stage in addition to *Streptomyces*, which is recognized as an important antibiotic producer.

Taken the shrimp-rearing water relationship into account, we discovered that some of the five most dominant bacterial genera mentioned previously were, in fact, common flora in both shrimp and rearing water samples. Some genera, however, were exclusive to either shrimp or their rearing water (Fig. S3a–d). For example, bacterial genera reported to be associated with phytoplankton such as *Neptuniibacter* (found in high abundance during the nauplius and zoea stages), *Altererythrobacter* (only found in dominance during the nauplius, zoea and mysis stages), *Roseibacterium* (found during the mysis stage), and *Marivita* (found during the postlarval stage) were determined entirely from the water samples (Fig. S3a). Moreover, *Vibrio* were only predominant in rearing water collected during the nauplius and zoea stages. Furthermore, *Tenacibaculum* was one of the most dominant genera within phylum *Bacteroidetes* determined from rearing water across all life stages with the exception of the mysis water sample (Fig. S3b). Stage-specific presence of some bacteria was also observed within this phylum. *Gilvibacter*, common marine bacteria, were abundant in shrimp and rearing water at the nauplius and zoea stages. On the contrary, *Phaeodactylibacter* existed in great numbers in rearing water but not in shrimp at the mysis and postlarval stages. Although *Planctomycetes* were found in a lower proportion in comparison to other phyla (Fig. 3a), the top five dominant genera were shared in shrimp and their rearing environments at all early life stages (Fig. S3c). *Ilumatobacter* (phylum *Actinobacteria*) was also found associated with shrimp and their rearing water samples from all stages (Fig. S3d). In contrast, *Microcella* was a dominant genus present in rearing environments spanning from nauplius to zoea stages, in which it was one of the top genera associated with zoeae shrimp. The shared genus distribution patterns between shrimp and their rearing water thus eluded to an evidence that environmental microbiota could potentially influence bacterial dynamics of the host shrimp.

### Comparison of the bacterial community structures from different stages

**Figure 4:**
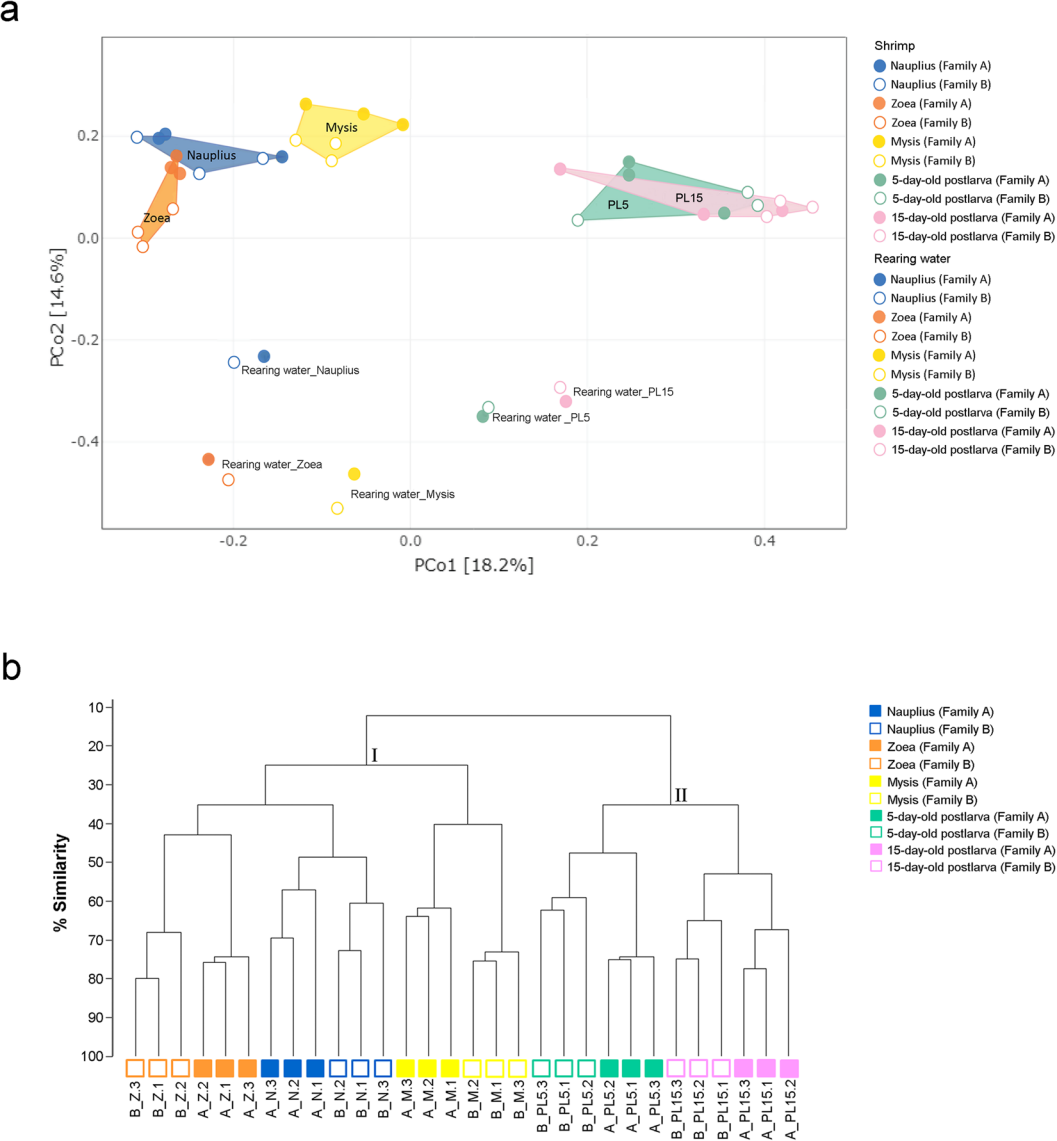
Principal coordinates analysis (PCoA) of bacterial communities associated in shrimp at different developmental stages (filled symbols) and rearing water (unfilled symbols) from Family A and Family B under different growth stages (**a**) and dendrogram of cluster analysis showing similarities in percent of OTUs in shrimp at different developmental stages. (**b**) Bacterial profiles were analyzed base on Bray-Curtis Dissimilarity method. Plot colors, blue, orange, yellow, green and pink represent nauplius (N), zoea (Z), mysis (M), 5-day-old postlarval (PL5) and 15-day-old postlarval (PL15) stages, respectively.

To compare the composition of bacteria associated with *P. monodon* at early developmental stages and rearing water, principal coordinates analysis (PCoA) based on the Bray-Curtis distance was used to assess dissimilarity at the OTUs level (Fig. 4a). Our results show that bacteria residing in host shrimp were clearly distinct from those found in rearing environments, indicating that bacteria patterns were specific to the animal host. Although the bacterial communities associated with shrimp were different across all four developmental stages, those corresponding to nauplius, zoea and mysis showed higher similar patterns to one another than those of the postlarval stage. Moreover, the bacterial profiles in PL5 and PL15 were not significantly different. Hence, our findings suggest that shrimp developmental stages had a substantial influence on the bacterial compositions which became more stable once shrimp had entered the postlarval stage. In accordance with the PCoA analysis, the percent similarity calculated based on Spearman’s correlation also revealed that bacterial profiles in postlarvae were distinctly clustered from those of the three early developmental stages (nauplius, zoea and mysis) (Fig. 4b). Within the same developmental stage, bacterial compositions in shrimp from the same family showed a higher similarity. The permutation multivariate analysis of variance (PERMANOVA) based on Bray-Curtis dissimilarity analysis was performed to determine the significance of association between different factors to microbiota^18^ (Table S2). According to the PERMANOVA analysis, we could confirm that shrimp developmental stages were strongly associated with bacterial profiles (*p* value = 0.001). The rearing environments also showed significantly different bacterial patterns from those associated to shrimp (*p* value < 0.05). Other factors, such as shrimp genetics (i.e. Family A and Family B), had comparatively minor or non-significant contributions to the bacterial composition associated with shrimp in their early life stages.

### Evidence of shared bacterial community associated with shrimp at early life stages

**Figure 5:**
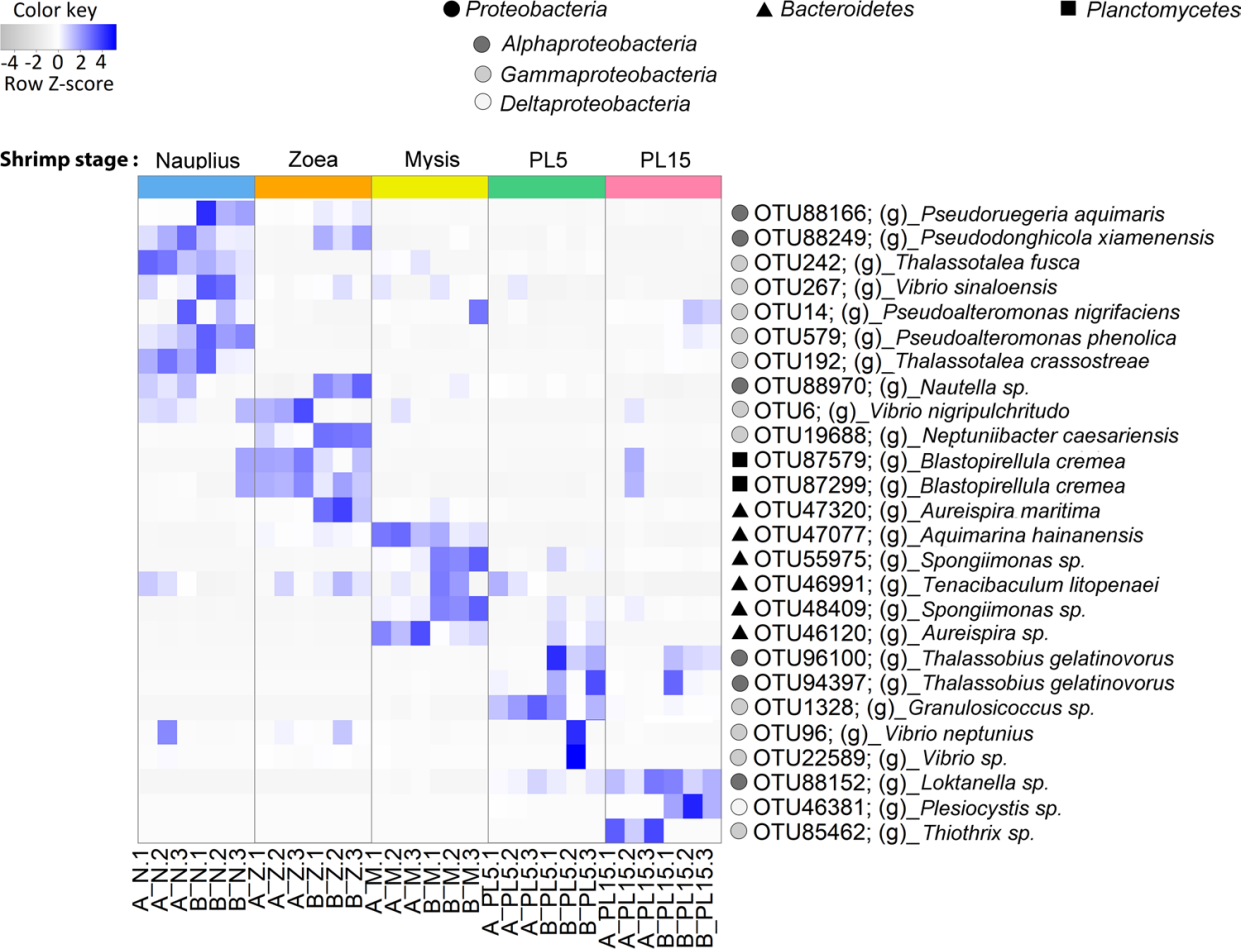
LEfSe analysis show differentially abundance OTUs of bacterial community-associated with early shrimp stages, which were divided to nauplius (N), zoea (Z), mysis (M), 5-day-old postlarval (PL5) and 15-day-old postlarval (PL15) from Family A and Family B. The threshold of the logarithmic linear discriminant analysis (LDA) score was 4.0. Circle, square and triangle, represent OTUs belong to *Proteobacteria*, *Planctomycetes*, and *Bacteroidetes*, respectively.

To determine the distribution of bacteria with respect to each developmental stage, bacterial relative abundance among early life stages was compared using the linear discriminant analysis effect size (LEfSe) tool with linear discriminant analysis (LDA) (Fig. 5). According to the LDA comparison, bacteria belonging to phyla *Proteobacteria*, *Bacteroidetes* or *Planctomycetes* had a different relative abundance in each life stage. For instance, bacteria belong to *Proteobacteria* were most identified in the nauplius, zoea and postlarval stages. In nauplius, the presence of *Pseudoruegeria*, *Pseudodonghicola*, *Thalassotalea*, *Vibrio* and *Pseudoalteromonas* was significant, while *Nautella*, *Vibrio*, *Neptuniibacter*, *Blastopirellula* and *Aureispira* were most abundant in zoea. *Blastopirellula* (phylum *Planctomycetes)* was also highly prevalent during this stage. Mysis shrimp showed a higher presence of *Aquimarina*, *Spongiimonas*, *Tenacibaculum* and *Aureispira* which belongs to phylum *Bacteroidetes*. Once entering the poslarval stage, shrimp in 5- and 15-day-old postlarva had similar bacterial diversity where the common bacterial genera included *Thalassobius*, *Granulosicoccus*, *Vibrio*, *Loktanella*, *Plesiocystis* and *Thiothrix*.

**Figure 6:**
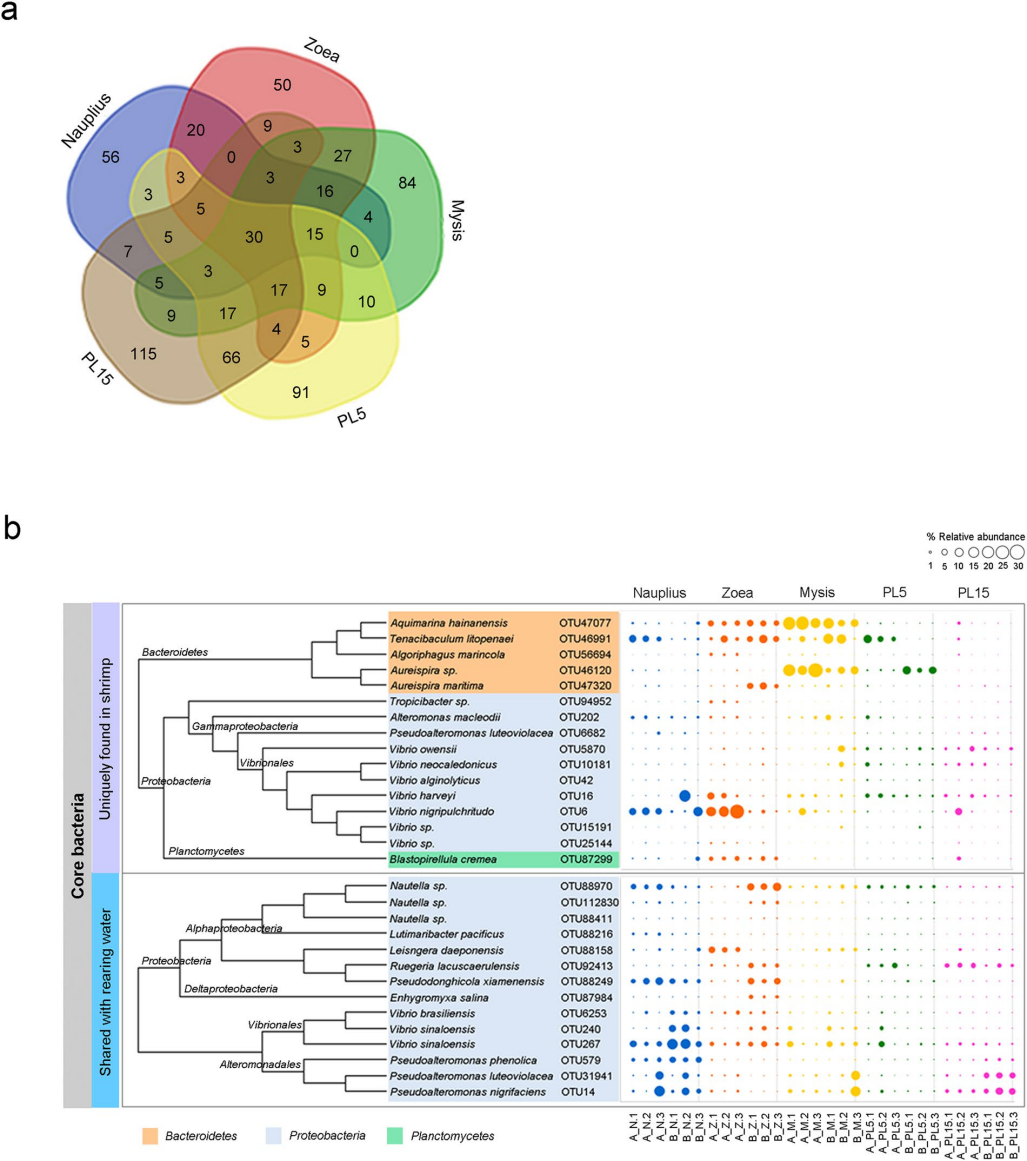
Venn diagram shows the numbers of OTUs that were unique or shared in shrimp at each life stage (**a**). The bubble plot represents relative abundance of unique or shared bacterial taxa at OTU level in shrimp at each life stages (nauplius, zoea, mysis, and 5-day-old postlarval (PL5) and 15-day-old postlarval (PL15) from Family A and B) and their rearing water.

To determine the common bacteria among the examined shrimp stages, a Venn diagram showed 30 shared OTUs that were associated with shrimp in all early developmental stages (Fig. 6a). Among these were 14 OTUs shared between shrimp and their environments that were affiliated mostly with *Proteobacteria*, such as *Nautella*, *Pseudodonghicola*, *Vibrio* and *Pseudoalteromonas* (Fig. 6b). Unique OTUs associated with shrimp were classified to phyla *Bacteroidetes*, *Proteobacteria* and *Planctomycetes* in which the dominant bacterial genera were *Aquimarina*, *Tenacibaculum*, *Aureispira* and *Vibrio*. Interestingly, *Tenacibaculum, Nautella, Pseudodonghicola, Alteromonas, Pseudoalteromonas*, and *Vibrio* were found in a high relative abundance in the nauplius stage and remained established thereafter, suggesting that they were commensal bacteria in early stages.

## Discussion

In recent years, there have been increasing pieces of evidence revealing the important roles of the host microbiome in digestion, enhancement of immune system and development in aquatic animals, including shrimp^10,19–21^. Hence, one of the main strategies to reach sustainable production and disease control in aquatic animals is to understand the host-microbial community relationship. Particularly, characterization of microbial establishment in their early life stages will be crucial due to their potential benefits to promote healthy gut microbial balance from young stages^22,23^. Our group has initiated efforts to investigate the bacterial community associated with *P. monodon* under various conditions^7–10,24^. Here, we present the first report on bacteria associated with early life stages in *P. monodon* from nauplius to zoea, mysis and postlarvae by using 16S rRNA gene sequencing analysis.

### The dynamic of bacteria associated with the early developmental stages

In this study, we reported the association of phyla *Proteobacteria*, *Bacteroidetes* and *Actinobacteria* with *P. monodon* at early life stages. These dominant phyla are known as typical microflora in various aquatic organisms. For instance, *Proteobacteria* and *Bacteroidetes* were distributed along the intestines of *P. monodon* juveniles^8,10,24^, *Litopenaeus vannamei*^25–28^ and zebrafish *Danio rerio*^29^. We also identified that *Vibrio* was the most dominant genus in all shrimp stages, which was congruent with other previous reports that it was commonly associated with various marine organisms^9,31^ and marine environment^30^. Furthermore, *Vibrio* has been reported as a dominant bacterium persisting through shrimp metamorphosis^32^. Within *Bacteroidetes, Tenacibaculum* was the most dominant, and has been reported to mutually exist with marine planktonic diatoms *Chaetoceros sp*. and *Thalassiosira sp*.^33^ which were fed to shrimp at the zoea and mysis stages in our study. Hence, a higher relative abundance of *Tenacibaculum* specifically during these two stages suggests that it could be a transient population. Additionally, *Planctomycetes* was identified as one of the dominant phyla during the early life stages by our findings. Although *Planctomycetes* was not as commonly reported widely in shrimp, this phylum was previously reported in *P. monodon* postlarvae^34^. Bacterial members in *Planctomycetes* have important roles in the aquaculture system by removing nitrogenous compounds, such as ammonia and nitrite^35^, that cause poison of fish or shrimp^36^.

It has been known that host factors are responsible for the establishment of microbiota in animals, including shrimp^37^. Here, we provide the evidence revealing that developmental stages were a major factor that affected the microbiota composition. While the bacterial communities of *P. monodon* associated with the postlarval stage (5- and 15-day old postlarvae) were similar, those from the earlier stages of nauplius, zoea and mysis were different. This drastic shift in the bacterial composition could be explained by the host physiology and change in diets required at each stage. Like other crustaceans, shrimp undergo metamorphosis through the following stages: egg, nauplius, zoea, mysis, postlarva, juvenile, and adult^38^. Progression of shrimp through their life stages also entails the difference in their physiological development and corresponding feed requirements^38,39^. In our study, when shrimp eggs hatched into the first larval stage (nauplius), they fed on their reserves for a few days before developing into the zoea stage where they were primarily fed with phytoplankton. Mysis shrimp were additionally fed with heat-treated brine shrimp *Artemia* besides phytoplankton. Once they entered the postlarval stage, their main diets comprised live *Artemia* and commercial feed pellets.

The shifts of bacterial communities across the developmental stages were also reported in other invertebrates, e.g. butterfly (*Heliconius erato*)^40^ and silkworm (*Bombyx mori*)^41^. Hence, the changes in both morphological and physiological development along with feed adjustments during the early life stages could cast an impact on the bacterial communities. In addition to developmental stages, several previous studies have highlighted that shrimp genetic background can also influence their bacterial composition^37,42^. In this study, we consistently observed that the bacterial profiles within the same family were clustered together in each life stage, suggesting that the genetic variation from different shrimp families could underlie the bacterial diversity observed in shrimp population, but the effect is not as substantial as host developmental stages. Our findings were coherent with the conclusions of past research that shed light on the direct relationship between shrimp developmental stages and microbiome^28,37,43^.

Apart from the animal host factors, environmental conditions can essentially regulate microbiome composition^44,45^, and have actually been shown to exert a stronger influence on animal microbiomes than host genetics^45,46^. Increasing pieces of evidence have highlighted a strong link between shrimp rearing environments (e.g. location, wild, farm and laboratory conditions) and microbial diversity^8,37,47^. Our study also demonstrated that the rearing environments could considerably shape the bacterial compositions in shrimp, as well as their developmental stages. Hence, microbial modulation through farming management, such as probiotics application and water condition, can be pursued to enrich specific microbiome composition as a feasible means to unlock a better growth performance or disease resistance.

### Shared microbiota associated with *P. monodon* at early developmental stages

Here, we provide the first evidence of microbiota of *P. monodon* established in their early life stages. The current study identified 30 common OTUs belonging to *Proteobacteria*, *Bacteroidetes* and *Planctomycetes*, suggesting the existence of bacterial establishment through *P. monodon* ontogeny. These common bacterial members were consistent with previous reports that *Proteobacteria* and *Bacteroidetes* were discovered as indigenous bacteria in juvenile shrimp^7–9^. Analysis of bacterial communities in growth developmental stages of human^4,48^ and thysanoptera^49^ revealed that the bacterial communities could be formed since the early life stages until they fully developed into adults, and continued to modulate the host’s immune system and enhance the protection against pathogen colonization and infection^50^. However, the host-bacteria interactions in *P. monodon* still require a much further investigation to obtain a better understanding of their relationships and potential benefits to the animal host.

The common bacteria could be earmarked as potential candidates for probiotic development as they are natural microflora that reside within the host, allowing for a sustainable administration and successful colonization in host shrimp. For instance, *Vibrio* and *Pseudoalteromonas* established since the nauplius stage in our study are commonly found in aquatic environments, and some strains have already been reported for a probiotic value against pathogenic bacteria^51,52^. Interestingly, *V. alginolyticus*, also identified among the shared microbiota by our analysis, has previously been proposed as a probiotic candidate^52^. Therefore, *Vibrio* might be utilized in the future as a potential probiotic for shrimp farming with great care being taken to ensure full benefits. This is because *V. harveyi*^10^ and *V. parahaemolyticus*^53^ might become pathogenic in shrimp under unfavorable conditions, such as high organic matter, poor farming management or immunocompromise of the host. Similarly, some species of *Pseudoalteromonas* are concerned as pathogenic bacteria^54^ while others have been used as probiotics in marine organisms including blue shrimp (*L. stylirostris*)^55^. However, further comparisons of microbiota associated with *P. monodon* at the early life stages obtained from different shrimp farms will be necessary to characterize the core microbiome shared by this shrimp species.

In conclusion, we have demonstrated that bacterial communities associated with *P. monodon* at the early life stages could be modulated by life stages through host physiological differences and diets. Our findings offer the first insights into bacterial establishment and assembly in the early life stages of *P. monodon* where the rearing environment plays minimal roles on influencing the shrimp microbiota. This fundamental knowledge will pave a way for sustainable development of probiotics or feed additives for aquaculture that will subsequently contribute to improved survival rates and nutrient absorption in *P. monodon* farming. Future research on host-microbiota interactions will be necessary to completely understand the dynamic of bacterial communities at different life stages and how they play roles on the shrimp’s well-being.

## Methods

### Shrimp and water sampling collection

The rearing of black tiger shrimp (*Penaeus monodon*) were carried at Shrimp Genetic Improvement Center (SGIC, National Science and Technology Development Agency, Surat Thani, Thailand). Seawater (salinity ~30ppt) used in the trial was pumped from the Gulf of Thailand, treated with ozone to remove potential pathogen contamination and stored in a water stocking tank. Each shrimp family was maintained in a 200-L fiberglass tank at a biosecure station and fed with diets based on their life stages. Briefly, shrimp at zoea stage were fed with microalgae *Thalassiosira sp*. and *Chaetoceros* sp. until they reached mysis. Mysis shrimp were fed with boiled *Artemia* and microalgae. Once reaching postlarval stage, they were fed with live *Artemia* in combination with commercial feed. Shrimp samples from each family were collected when they reached nauplius, zoea, mysis, and postlarvae stages (Fig. 1). Due to small sizes of shrimp larvae, shrimp were pooled together, and each pooled shrimp sample contained triplicate. All shrimp samples were washed twice in sterile distilled water to minimize carried-over contamination from rearing water. For water sample, 50 mL of rearing water were sampling from three random locations per tank. The rearing water from the same shrimp stages were pooled into a total of 150 mL and filtered through a 0.22 µm Mixed cellulose esters membrane filter (Millipore, Ireland). Both shrimp and filter membranes were stored at −80 °C until used.

### DNA extraction and purification

Each pooled shrimp sample was carefully ground in a mortar containing liquid nitrogen. An equal amount of 50 mg from each ground tissue sample was applied for DNA extraction by using QIAamp DNA Mini Kit (Qiagen, Germany), and performed according to the manufacturer’s instruction. For water samples, the filter membrane was excised to small pieces, placed in the 2 mL tube containing extraction buffer from the kit and vortexed until the membrane was cleared. All DNA samples were treated with proteinase K following by RNase A. DNA samples were further purified and concentrated by using Genomic DNA Clean and Concentrator (Zymo Research, USA) according to the manufacturer’s instruction. The DNA purity and concentration were determined by NanoDrop (ND-8000) spectrophotometer. The DNA was stored at −20 °C until further used.

### rRNA gene amplification and sequencing

The 16S rRNA fraction containing the V3-V4 region was amplified with the primer pairs containing sequencing adapters (italic) 16S MiSeq F (5′ *TCG TCG GCA GCG TCA GAT GTG TAT AAG AGA CAG* CCT ACG GGN GGC WGC AG 3′) and 16S MiSeq R (5′ *GTC TCG TGG GCT CGG AGA TGT GTA TAA GAG ACA G*GA CTA CHV GGG TAT CTA ATC C 3′)^56^. The 16S rRNA gene amplification was performed using a proofreading Q5 High-Fidelity DNA polymerase (New England Biolabs, USA) with following PCR parameters; initial denaturation at 98 °C for 3 min, and 25 cycles of 98 °C for 30 s, 54 °C for 30 s and 72 °C for 30 s. The final extension was carried out at the 72 °C for 2 min. The quality of PCR amplicons was analyzed on 1.5% agarose gel electrophoresis and purified by using QIAquick Gel Extraction Kit (Qiagen, Germany) according to the supplier’s standard instruction. The quality and quantity of 16S rRNA amplicons were determined by NanoDrop (ND-8000) spectrophotometer and visualized on 1.5% agarose gel electrophoresis. The 16S amplicon libraries were submitted for Illumina sequencing at Macrogen Inc. (Korea).

### Quantification of bacterial abundance using real-time PCR analysis

Primer pairs specific for all bacteria (Eub338 and Eub518), *Alphaproteobacteria* (Eub338 and Alf685R, *Gammaproteobacteria* (1080γF and γ1202R), *Firmicutes* (Lgc353 and Eub518), *Actinobacteria* (Act920F3 and Act 1200 R) and *Bacteroidetes* (798CfbF and Cfb967R)^57,58^. Each of a 10 µL reaction contained a DNA template (100 ng for shrimp and 1 ng for water), 0.2 µM of each primer and 1X SYBR Green supermix (BioRad). The PCR parameters were initial denaturation at 95 °C for 3 min, followed by 40 cycles of 95 °C for 30 s, 54 °C for 20 s and 72 °C for 30 s. The specificity of each PCR product was confirmed by melting curve analysis performed from 55 °C to 95 °C with a continuous fluorescent reading with a 0.5 °C increment. To determine copy numbers, a standard curve was constructed using 10-fold serial dilutions of plasmid DNA and the target copy number was calculated from the standard curve equation. The relative abundance for each target bacteria was determined by normalizing with the abundance of total bacteria in that sample and percent relative abundance was determined within five target taxa.

### amplicon sequence data processing

The 300 paired-end Illumina MiSeq DNA reads were cleaned-up by removing adapters and low-quality bases (phred score ≤ 20) using TrimGalore (http://www.bioinformatics.babraham.ac.uk/projects/trim_galore/). Low-quality reads including the reads shorter than 150 nucleotides and the reads with homopolymers> 6 were filtered out. The read quality was confirmed after the clean-up process by using FastQC^59^ and MultiQC^60^. The sequencing reads were classified into taxonomic order by using MOTHUR version 1.39.5^61^ according to MiSeq standard operation procedure^62^. Sequences with 97% identity threshold were clustered into operational taxonomic units (OTUs) and classified at a confidence threshold of 80 using the custom taxonomic reference database. The custom taxonomic reference database was generated based on 33 phylum nomenclature in the List of Prokaryotic Names with Standing in Nomenclature (LPSN)^63^. The bacterial sequences which their phylum nomenclature is listed on the LPSN were extracted from version 16 of the Ribosomal database project (RDP)^64^. Unclassified OTUs to bacterial taxonomy and those contain only one read across all samples were filtered out before downstream analysis.

### Microbial community analysis

The microbial diversity was assessed using rarefaction and alpha diversity indices (Chao1 and Shannon) according to OTU (equivalent to species level) by using vegen^65^ and phyloseq.^66^ package in R^67,68^. Diversity indices were calculated using rarefied to an even depth of 33,269 read per sample. Good’s coverage was calculated by using the following formula: Coverage = 1 − (n/N), where n is the number of singletons and N is the total number of observed OTUs^69,70^. Microbiome variation within and between each condition were assessed using a permutational ANOVA (PERMANOVA; Adonis) based on a Bray-Curtis dissimilarity matrix of the taxonomic profile in the vegan package^65^. Statistical analysis of bacterial diversity in early life stages was performed using ANOVA with a post-hoc (Duncan’s multiple rank test) in SPSS. Difference in microbial composition was visualized in two-dimensional space using principal coordinates analysis (PCoA) with Bray-Curtis distance by using Ampvis package in R^71^. To characterize the bacterial community, linear discriminant analysis effect size (LEfSe) on galaxy platform (http://huttenhower.sph.harvard.edu/galaxy/) was employed to identify different microbial community in early life stages of shrimp. Furthermore, the shared microbiome was defined as the bacterial OTUs that presented in all samples from either nauplius or zoea until 15-day-old postlarva. Since taxonomy assignment using RDP database in Mothur can classify only at genus level, the OTUs that were identified by using LEfSe and shared microbiome analysis were re-classified at species-level to provide in depth information. Taxonomy nomenclature of OTUs was further assigned by using the BLAST and consensus taxonomy classifier against the 16S ribosomal RNA sequences (Bacteria)^72^.

## Supplementary information

Supplementary information.

## Data Availability

The datasets of 16S rRNA amplicon sequences were deposited to BioProject at NCBI under accession number PRJNA540737.
