# Supplementary material for: Bacterial analysis in the early developmental stages of the black tiger shrimp (Penaeus monodon)
Source: Sci Rep. 2020 Mar 17;10:4896. doi: 10.1038/s41598-020-61559-1 (PMC7078212; doi:10.1038/s41598-020-61559-1)

Bacterial analysis in the early developmental stages of the black tiger shrimp (*Penaeus monodon*),

Pacharaporn Angthong

Tanaporn Uengwetwanit

Sopacha Arayamethakorn

Panomkorn Chaitongsakul

Nitsara Karoonuthaisiri

Wanilada Rungrassamee

Table S1. Sampling depth analysis of the black tiger shrimp at early developmental stages and rearing water (W) during shrimp cultivation, which were collected at the stages of nauplius (N), zoea (Z), mysis (M), 5-day-old postlarva (PL5) and 15-day-old postlarva (PL15) from Family A and Family B. Sequences were clustered into operational taxonomic units (OTUs) at 97% similarity.

Stage	Family	Sample ID	OTUs	Phylum	Class	Order	Family	Genus	Good's coverage
Nauplius (N)	A	N.1	2,509	11	24	60	127	310	0.86
		N.2	711	9	16	41	75	173	0.99
		N.3	629	10	17	35	63	155	1.00
		Average	1,283	10	19	45	88	213	0.95
	B	N.1	2,343	13	22	52	113	263	0.84
		N.2	2,058	10	20	52	106	246	0.84
		N.3	3,433	14	27	59	124	314	0.84
		Average	2,611	12	23	54	114	274	0.84
Zoea (Z)	A	Z.1	2,290	13	24	54	105	254	0.85
		Z.2	2,114	12	23	48	91	226	0.87
		Z.3	2,533	12	24	54	101	247	0.85
		Average	2,312	12	24	52	99	242	0.86
	B	Z-1	3,230	11	21	49	105	256	0.83
		Z-2	738	8	14	31	46	124	0.99
		Z-3	2,645	10	18	45	96	243	0.84
		Average	2,204	10	18	42	82	208	0.89
Mysis (M)	A	M.1	2,308	13	26	60	122	287	0.85
		M.2	3,002	12	19	44	88	211	0.84
		M.3	2,429	9	17	44	85	220	0.85
		Average	2,580	11	21	49	98	239	0.85
	B	M.1	2,579	10	17	43	89	206	0.82
		M.2	2,384	10	18	41	81	199	0.83
		M.3	3,346	11	19	48	102	241	0.82
		Average	2,770	10	18	44	91	215	0.82
5-day-old postlarva (PL5)	A	PL5.1	2,850	10	20	54	114	290	0.85
		PL5.2	3,349	11	23	55	114	309	0.85
		PL5.3	2,775	10	22	55	115	278	0.83
		Average	2,991	10	22	55	114	292	0.84
	B	PL5-1	2,680	11	22	51	105	274	0.83
		PL5-2	2,397	10	21	52	100	235	0.88
		PL5-3	767	10	20	43	75	175	1.00
		Average	1,948	10	21	49	93	228	0.90
15-day-old postlarva (PL15)	A	PL15.1	2,180	9	18	48	99	242	0.88
		PL15.2	2,805	11	21	55	122	301	0.88
		PL15.3	3,223	12	26	59	123	305	0.87
		Average	2,736	11	22	54	115	283	0.88
	B	PL15.1	661	10	17	36	61	145	0.99
		PL15.2	2,727	10	20	46	100	260	0.87
		PL15.3	2,818	11	19	44	94	262	0.86
		Average	2,069	10	19	42	85	222	0.91
		Average±S.D. (all stages)	2,350±835	11±1	21±3	49±8	98±20	242±51	0.87±0.06

**Stage**	**Family**	**Sample ID**	**OTUs**	**Phylum**	**Class**	**Order**	**Family**	**Genus**	**Good's coverage**
Rearing water (W)	A	N	2,791	15	32	74	173	437	0.86
	B	N	3,080	14	29	70	157	433	0.86
	A	Z	2,728	10	20	48	105	273	0.82
	B	Z	2,334	10	19	49	96	250	0.82
	A	M	2,052	10	20	46	98	249	0.85
	B	M	2,005	10	21	47	101	242	0.83
	A	PL5	3,177	13	24	57	131	359	0.85
	B	PL5	2,573	11	22	57	125	318	0.85
	A	PL15	2,847	10	22	53	114	305	0.84
	B	PL15	3,111	10	22	54	119	316	0.83
		Average±S.D. (all stages)	2,670±424	11±2	23±4	56±10	122±26	318±72	0.84±0.02

Table S2 Statistical output for Permutational ANOVA (PERMANOVA; adonis) analyses of microbiome composition

**Factor**		***F* value**	**R^2**	***p* value**
**Developmental stages (Shrimp)**		3.323	0.347	0.001
**Host vs rearing environment**				
	Nauplius	2.866	0.323	0.047
	Zoea	1.889	0.239	0.043
	Mysis	2.649	0.306	0.030
	PL5	2.402	0.286	0.027
	PL15	2.539	0.297	0.038
**Host genetics (Family A vs B)**				
	Nauplius (Family A vs B)	1.763	0.306	0.100
	Zoea (Family A vs B)	2.034	0.337	0.100
	Mysis (Family A vs B)	1.923	0.325	0.100
	PL5 (Family A vs B)	1.963	0.329	0.100
	PL15 (Family A vs B)	1.672	0.295	0.100

Fig. S1

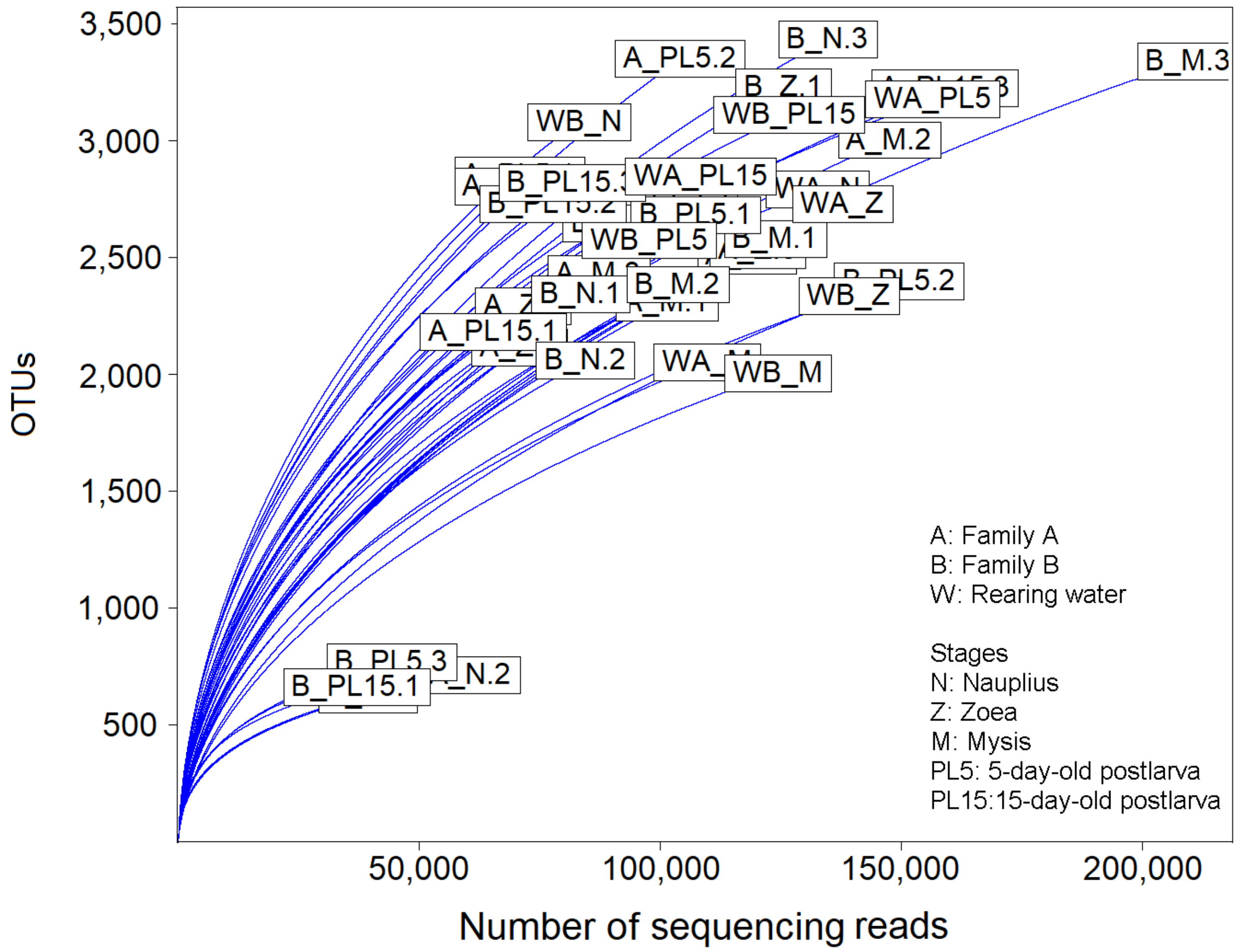
OTUs
Number of sequencing reads
A: Family A
B: Family B
W: Rearing water
Stages
N: Nauplius
Z: Zoea
M: Mysis
PL5: 5-day-old postlarva
PL15:15-day-old postlarva
B_M.3
B_N.3
A_PL5.2
WB_N
WA_PL5
WB_PL15
A_M.2
WA_PL15
WA_Z
B_PL5.1
WB_PL5
B_M.1
B_M.2
B_N.1
WB_Z
A_PL15.1
B_N.2
WB_M
B_PL15.1

Fig. S2

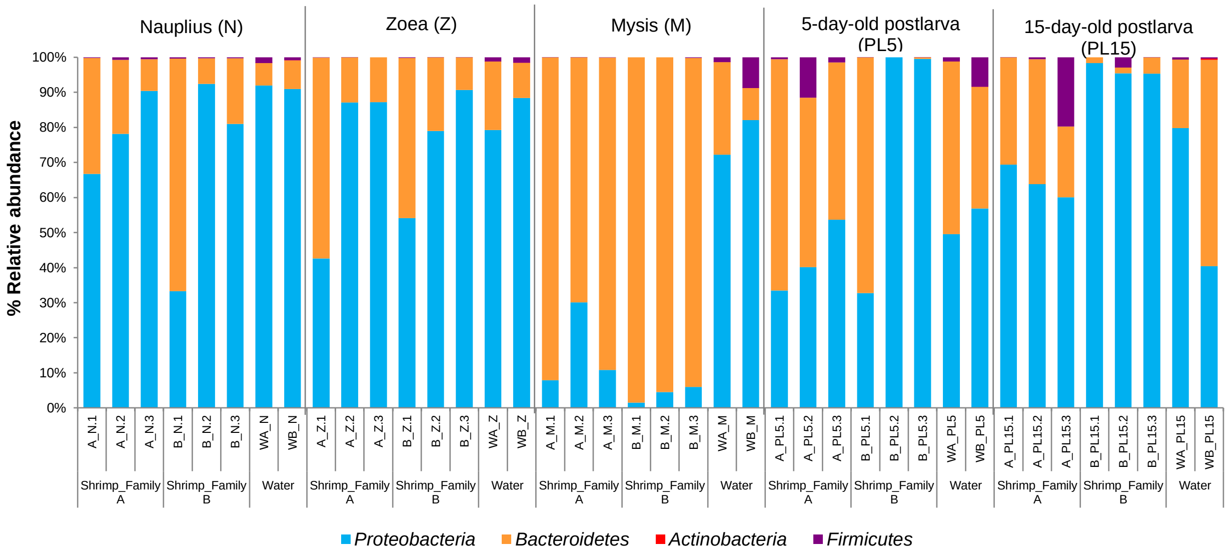
Nauplius (N)
Zoea (Z)
Mysis (M)
5-day-old postlarva (PL5)
15-day-old postlarva (PL15)
% Relative abundance
100%
90%
80%
70%
60%
50%
40%
30%
20%
10%
0%
A_N.1 A_N.2 A_N.3 B_N.1 B_N.2 B_N.3 WA_N WB_N
A_Z.1 A_Z.2 A_Z.3 B_Z.1 B_Z.2 B_Z.3 WA_Z WB_Z
A_M.1 A_M.2 A_M.3 B_M.1 B_M.2 B_M.3 WA_M WB_M
A_PL5.1 A_PL5.2 A_PL5.3 B_PL5.1 B_PL5.2 B_PL5.3 WA_PL5 WB_PL5
A_PL15.1 A_PL15.2 A_PL15.3 B_PL15.1 B_PL15.2 B_PL15.3 WA_PL15 WB_PL15
Shrimp_Family A
Shrimp_Family B
Water
Proteobacteria
Bacteroidetes
Actinobacteria
Firmicutes

Fig. S3

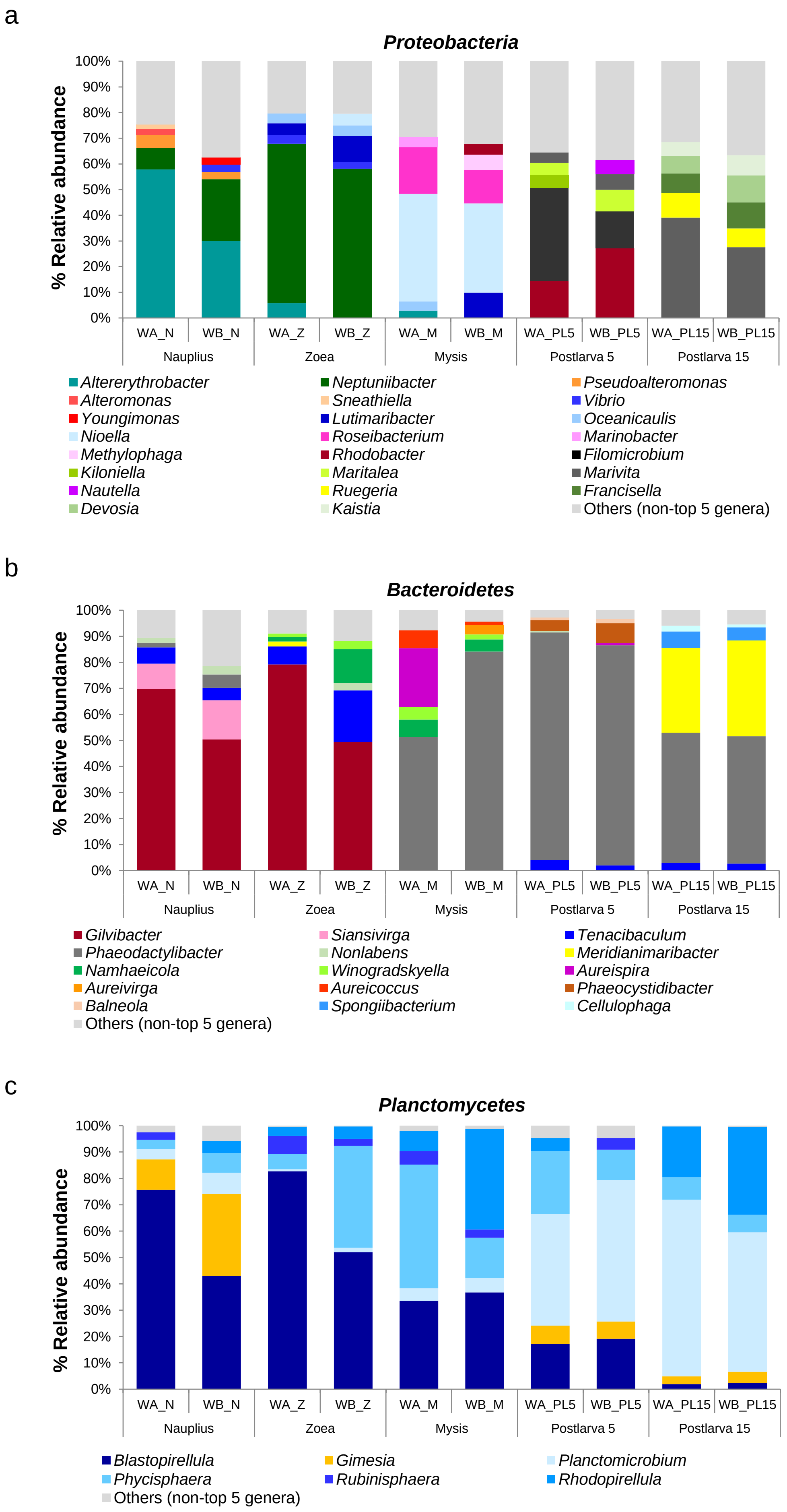
a
Proteobacteria
% Relative abundance
100%
90%
80%
70%
60%
50%
40%
30%
20%
10%
0%
WA_N WB_N WA_Z WB_Z WA_M WB_M WA_PL5 WB_PL5 WA_PL15 WB_PL15
Nauplius Zoea Mysis Postlarva 5 Postlarva 15
Altererythrobacter
Neptuniibacter
Pseudoalteromonas
Alteromonas
Sneathiella
Vibrio
Youngimonas
Lutimaribacter
Oceanicaulis
Nioella
Roseibacterium
Marinobacter
Methylophaga
Rhodobacter
Filomicrobium
Kiloniella
Maritalea
Marivita
Nautella
Ruegeria
Francisella
Devosia
Kaistia
Others (non-top 5 genera)
b
Bacteroidetes
% Relative abundance
100%
90%
80%
70%
60%
50%
40%
30%
20%
10%
0%
WA_N WB_N WA_Z WB_Z WA_M WB_M WA_PL5 WB_PL5 WA_PL15 WB_PL15
Nauplius Zoea Mysis Postlarva 5 Postlarva 15
Gilvibacter
Siansivirga
Tenacibaculum
Phaeodactylibacter
Nonlabens
Meridianimaribacter
Namhaeicola
Winogradskyella
Aureispira
Aureivirga
Aureicoccus
Phaeocystidibacter
Balneola
Spongiibacterium
Cellulophaga
Others (non-top 5 genera)
c
Planctomycetes
% Relative abundance
100%
90%
80%
70%
60%
50%
40%
30%
20%
10%
0%
WA_N WB_N WA_Z WB_Z WA_M WB_M WA_PL5 WB_PL5 WA_PL15 WB_PL15
Nauplius Zoea Mysis Postlarva 5 Postlarva 15
Blastopirellula
Gimesia
Planctomicrobium
Phycisphaera
Rubinisphaera
Rhodopirellula
Others (non-top 5 genera)

Fig. S3 (continued)

d

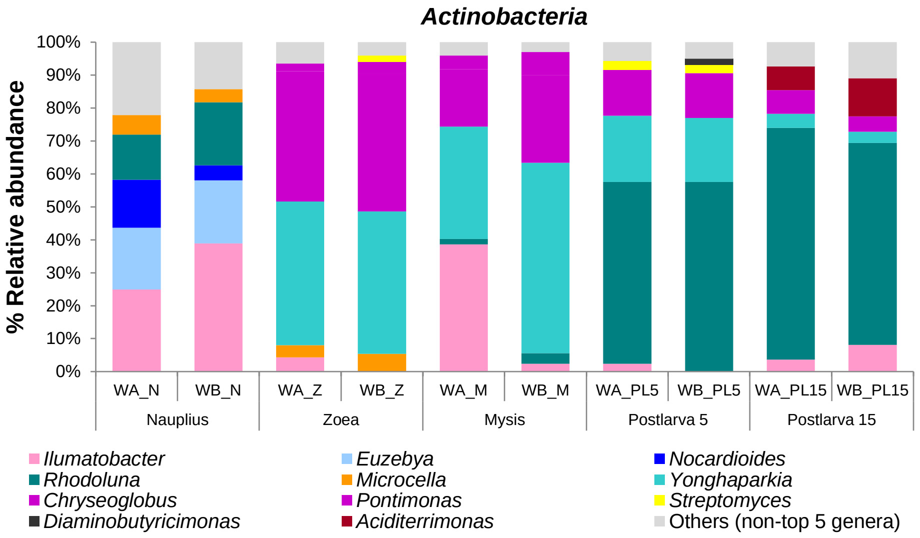

Supplement: Supplementary file 1 — Supplementary information. [file 41598_2020_61559_MOESM1_ESM.pdf]
